# Growth and fabrication of InAs/GaSb type II superlattice mid-wavelength infrared photodetectors

**DOI:** 10.1186/1556-276X-6-635

**Published:** 2011-12-22

**Authors:** Jianxin Chen, Qingqing Xu, Yi Zhou, Jupeng Jin, Chun Lin, Li He

**Affiliations:** 1 Key Laboratory of Infrared Imaging Materials and Detectors, Shanghai Institute of Technical Physics, Chinese Academy of Sciences, Shanghai, 200083, China

**Keywords:** InAs/GaSb, type II superlattice, photodiodes, infrared

## Abstract

We report our recent work on the growth and fabrication of InAs/GaSb type II superlattice photodiode detectors. The superlattice consists of 9 monolayer InAs/12 monolayer GaSb in each period. Lattice mismatch between the GaSb substrate and the superlattice is 1.5 × 10^-4^. The full width at half maximum of the first-order satellite peak from X-ray diffraction is 28 arc sec. The P-I-N photodiodes in which the absorption regions (I regions) have 600 periods of superlattice show a 50% cutoff wavelength of 4.3 μm. The current responsivity was measured at 0.48 A/W from blackbody radiation. The peak detectivity of 1.75 × 10^11 ^cmHz^1/2^/W and the quantum efficiency of 41% at 3.6 μm were obtained.

**PACS: **85.60.-q; 85.60.Gz; 85.35.-Be.

## Introduction

HgCdTe [MCT] photodetectors which offer excellent quantum efficiency are the dominating infrared technology, and very large MCT sensor arrays are available. The drawbacks of MCT come from its technology difficulty. MCT has weak mechanical strength due to the weak ionic bonds and low uniformity due to the high Hg vapor pressure. Common substrates for MCT epitaxial growth are lattice-matched CdZnTe or readily available Si or Ge capped with a few-micron-thick buffer layers, yet no substrates are known to date which can satisfy all necessities for being low cost, lattice-matched, and chemically, mechanically, and optically well suited. Thus, the fabrication of large-format MCT arrays with homogeneous performances becomes more and more challenging, especially with an increasing cutoff wavelength. Alternative approaches based on quantum, mechanically tailored semiconductor heterostructures have been developed including quantum well infrared photodetectors, quantum dot infrared photodetectors, and InAs/GaSb type II, strained-layer superlattice [SLS] photodetectors.

The idea of the InAs/GaSb superlattice [SL] was firstly introduced by Sai-Halasz et al. in 1977 [1]. It was proposed to be used for infrared detection by Smith and Mailhoit in 1987 [2]. Since then, the InAs/GaSb SL has received more and more attentions for infrared detection due to its unique advantages over other infrared materials. The effective bandgap of the SL material can be tailored over a wide range (3 μm ≤ *λ *≤ 30 μm) by varying the thickness of InAs and GaSb, the two 'mid-gap' constituent materials. Auger recombination is suppressed due to large splitting between the heavy-hole and light-hole subbands induced by the strains in the SL. Tunneling currents in SL detectors are reduced due to the large electron effective mass. The band structures of the SL material can be engineered to enhance carrier lifetimes and reduce noises. InAs/GaSb SL detectors are actually the only candidate that has theoretically predicted performances better than MCT [3-5]. Moreover, the type II SLs have the advantages of excellent uniformity and low cost due to the mature III-V material technology. The first high-performance InAs/GaSb photodetector was demonstrated in 1996 [6]. The first SL focal plane array [FPA] detector was reported in 2003 [7]. Some leading laboratories have recently demonstrated megapixel FPA detectors [8,9].

Despite the rapid progress, InAs/GaSb type II SL photodetectors are still at their infancy time. Their performances have far not reached their theoretical prediction yet and are even inferior to those of MCT. One important issue which limited the performances of SLS detectors is the SL material quality [10]. Yang et al. reported that the carrier lifetimes of SLS are limited by the Shockley-Read-Hall recombination when the carrier concentration is lower than 10^17 ^cm^-3 ^[11]. Therefore, it is desired to grow high-quality SL materials with high crystal perfection for device applications. Rodriguez et al. reported their mid-wavelength infrared SL materials with an X-ray diffraction full width at half maximum [FWHM] of 26 arc sec [12]. Khoshakhlagh et al. [13] reported a FWHM of about 32 arc sec for a SL material with an 8-μm cutoff wavelength. In SL material growth, the control of interface type and quality dramatically affects the overall material quality [13]. As InAs and GaSb have no common atoms and both arsenic and antimony's sticking coefficients are less than 1, two types of interfaces may be formed, the GaAs-like and the InSb-like, according to the growth conditions. Therefore, in InAs/GaSb SL growth, the interface controls are particularly complicated and important.

We report in this study the material growth and device fabrication of InAs/GaSb SL mid-infrared photodiodes. In particular, we designed a new shutter sequence in the growth process to improve the interface quality. This is our first effort to bring up the InAs/GaSb material technology for infrared detection in our laboratory.

## Material growth and device fabrication

The InAs/GaSb SL is grown by molecular beam epitaxy [MBE] on n-type doped (100) GaSb substrates. The growth temperature was set at 450°C and a V/III ration of 5:1. There are two important issues uniquely associated with InAs/GaSb SL growth: (1) InAs has 0.75% smaller lattice constant than GaSb, and proper interface layers, in typical InSb, have to be inserted between the InAs and GaSb layers for strain balance and (2) interface control is extremely important to obtain high-quality epitaxial materials since there are no common atoms between InAs and GaSb. Shutter sequences were carefully designed to favor an InSb-like interface layer with a desired thickness. InSb-like interfaces were realized through proper shutter sequences. An important issue is to suppress the arsenic flux at the interface growth since arsenic has a high background pressure in a MBE chamber. At GaSb-to-InAs interfaces, we first closed the gallium cell shutter, left the antimony cell shutter open for 2 s, and then opened the indium cell for 0.8 s before opening the arsenic shutter for the InAs growth. At the InAs-to-GaSb interface, we first closed the arsenic shutter and left the indium shutter open for 1 s to have the growing surface covered by one layer of indium atoms, then closed the indium shutter, and opened the antimony shutter to switch to the GaSb growth. With the above shutter sequence, we greatly reduced the arsenic background pressure during the InSb and GaSb growth. The SL crystal qualities, such as the period and the lattice-mismatch from the GaSb substrates, were characterized by high-resolution X-ray diffraction [HRXRD]. The surface quality was measured by atomic force microscopy [AFM].

**Figure 1:**
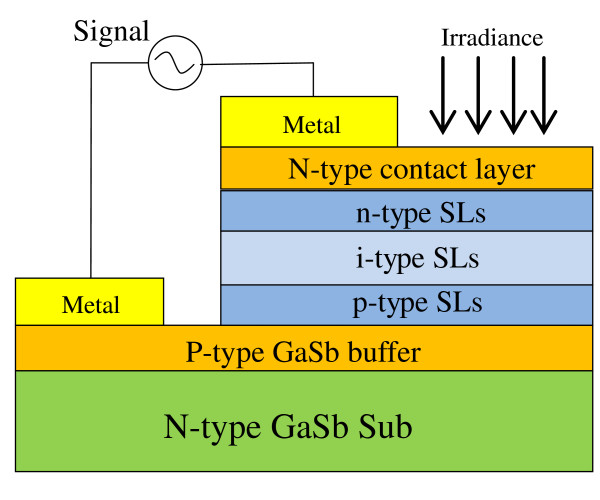
**The schematic cross section profile of a single-element detector**. The detectors are designed to receive the irradiance from the front side in order to avoid the strong GaSb substrate absorption.

The photodetector has a P-I-N structure. It consists of 320-nm-thick p-type SLs with a 10^17^-cm^-3 ^Be doped in the GaSb layer, 3.2-μm-thick undoped i-type SLs, and 320-nm-thick n-type SLs with a 1.5 × 10^17 ^cm^-3 ^Si doped in the InAs layer. All the SLs consist of 9 monolayer [ML] InAs and 12 ML GaSb. A 1-μm-thick GaSb layer with Be doping to an order of 5 × 10^17 ^cm^-3 ^and a 30-nm-thick InAs layer with Si doping to an order of 5 × 10^17 ^cm^-3 ^were below and above the P-I-N structure, respectively, as a contact layer. The single-element detectors have architecture as shown in Figure 1. The detectors are designed to receive the irradiance from the front side in order to avoid the strong GaSb substrate absorption [10].

**Figure 2:**
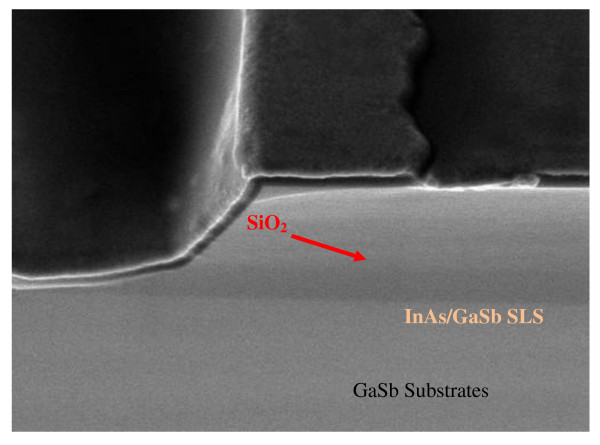
**A SEM showing the sidewall passivation of the detector**. The whole sidewall of the mesa was protected by the SiO_2 _layer.

Contact photolithography was employed for device fabrication. The mesas were wet etched with a mixed solution of citric acid, phosphoric acid, and hydrogen peroxide (10:1:1). The sidewall of the mesa is then passivated by sputtering a 300-nm-thick SiO_2 _layer. Figure 2 shows a scanning electron micrograph [SEM] of an etched and passivated detector mesa. Contact windows were then opened by inductively coupled plasma reactive-ion etching. A composite contact layer of 20-nm Ti, 30-nm Pt, and 20-nm Au was deposited by electron-beam evaporation on both the p-type GaSb buffer layer and the n-type top InAs layer. It was followed by thermal vapor evaporation of a thick Au layer for wire bonding. Finally, single-element devices of different photosensitive areas varying from 100 μm × 100 μm to 500 μm × 500 μm were fabricated.

The detectors were mounted onto homemade cold fingers in a Dewar, which were cooled to 77 K with liquid nitrogen. Spectral responsivity was measured using Fourier transform infrared [FTIR] spectroscopy. The current-voltage and dynamic resistance [DR]-voltage curves were swept by a Keithley 236 source-measure unit (Keithley Instruments, Inc., Shanghai, China) using a self-coded LabVIEW program. For photoresponse measurements, the blackbody temperature was set at 800 K, and the chopper, at 800 Hz. The signals were picked up by a preamplifier and a lock-in amplifier.

## Results and discussion

**Figure 3:**
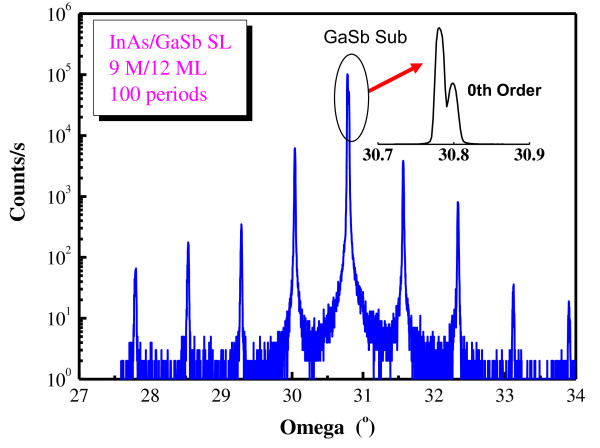
**The ω-2Θ scanning curve of an InAs/GaSb SL structure**. The curve was measured by HRXRD. The SL was designed to consist of 12 ML GaSb and 9 ML InAs in each period.

Figure 3 shows a high-resolution ω-2Θ scanning curve of a 9 ML InAs/12 ML GaSb SL. The layer numbers of InAs and GaSb in each period were determined to achieve a 4.5-μm cutoff wavelength using a k·p model under the envelope function approximation. The SL consists of 100 periods. Clear and sharp satellite peaks up to the fourth order are observed. Higher-order peaks could be observed if we had extended the scanning angle range. The measured lattice mismatch between the SL and the GaSb substrate is Δa/a = 1.5 × 10^-4^. The FWHM of the first-order peak is 28 arc sec. The thickness of each period is 65.3 Å according to the simulation. It has only a 2% difference comparing with the design thickness (63.8 Å).

**Figure 4:**
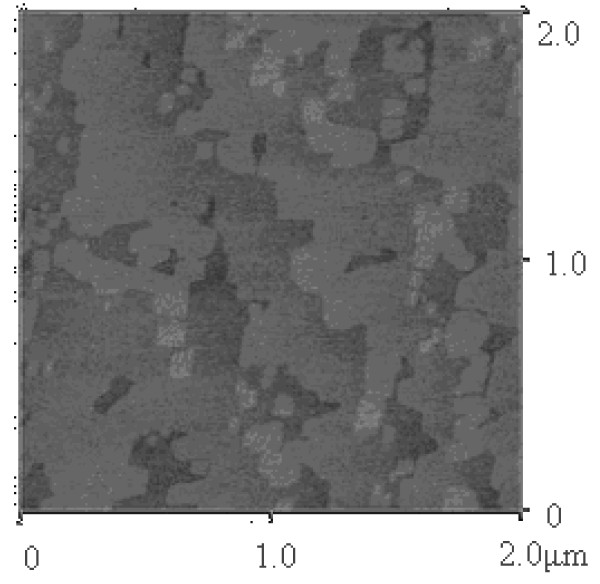
**An AFM topographic scan of an InAs/GaSb SL sample**. The measured area is 2 μm × 2 μm. Clear atomic stages can be observed in the graph.

The surface morphology was studied with a Digital Instruments Nanoscope atomic force microscope (Santa Barbara, CA, USA) as shown in Figure 4. Atomic steps are clearly observed. We have achieved a root-mean-square roughness of 1.5 Å over an area of 2 μm × 2 μm.

**Figure 5:**
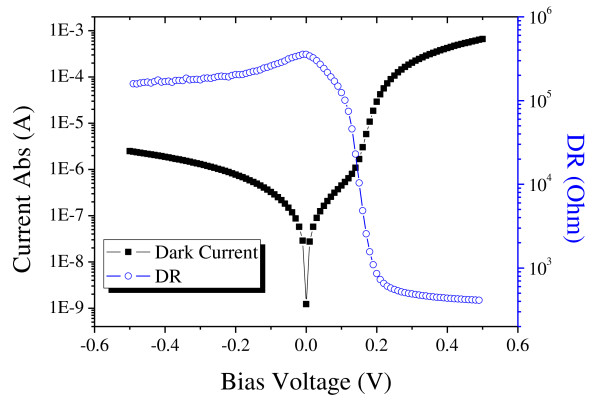
**Dark current and DR of a photodiode with a 200 μm × 200 μm area**. The R_0_A of the detector is 147 Ω cm^2^.

**Figure 6:**
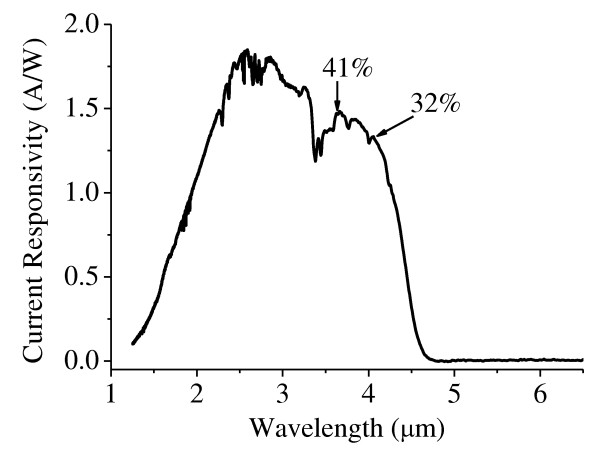
**The current responsivity spectrum of a SL photodetector**. The 50% cutoff wavelength is 4.3 μm. The numbers labeled in this figure indicate the quantum efficiency, which is 41% at a wavelength of 3.6 μm.

Figure 5 shows the measured dark current curve and the corresponding DR curve of a 200 μm × 200 μm photodiode. For this photodiode, a resistance-area product at zero bias [R_0_A] of 147 Ω cm^2 ^was measured. A current responsivity of 0.48 A/W was measured for the same device, and it has a blackbody detectivity of 4.54 × 10^10 ^cmHz^1/2^/W. The InAs/GaSb SL detectors have a 50% cutoff wavelength of 4.3 μm at a longer wavelength side and 2.0 μm at a shorter wavelength side according to the FTIR spectroscopy. Combining the response spectrum and the blackbody current responsivity, the absolute current responsivity spectrum and quantum efficiency can be calculated. The result is shown in Figure 6. The numbers labeled in the figure represent the quantum efficiency. The *G *factor was calculated to be 0.26, and the peak detectivity reaches to 1.75 × 10^11 ^cmHz^1/2^/W.

## Conclusions

In summary, we grew and fabricated InAs/GaSb type-II SL materials and devices by MBE and wet chemical etching. Lattice mismatch between the substrate and the SL is 1.5 × 10^-4^. The FWHM of the first-order satellite peak from XRD is 28 arc sec. The mid-wavelength infrared photodiodes have a cutoff wavelength of 4.3 μm. A current responsivity of 0.48 A/W and a peak detectivity of 1.75 × 10^11 ^cmHz^1/2^/W were measured. The quantum efficiency of the device at 3.6 μm is 41%.

## Competing interests

The authors declare that they have no competing interests.

## Authors' contributions

JC contributed the main ideas of SL structure design and supervised the MBE growth. QX carried out the MBE growth. YZ carried out the SL X-ray measurements. JJ carried out the device processing. CL supervised the device processing and carried out the device measurements. LH initiated and supervised the SL infrared detector program. All authors read and approved the final manuscript.

## References

[B1] Sai-Halasz GA Tsu R Esaki L A new semiconductor superlattice Appl Phys Lett 1977 30 651 653 10.1063/1.89273

[B2] Smith DL Mailhiot C Proposal for strained type II superlattice infrared detectors J Appl Phys 1987 62 2545 2548 10.1063/1.339468

[B3] Grein CH Young PM Flatte ME Ehrenreich H Long wavelength InAs/GaSb infrared detectors: optimization of carrier lifetimes J Appl Phys 1995 78 7143 7152 10.1063/1.360422

[B4] Youngdale ER Meyer JR Hoffman CA Bartoll FJ Auger lifetime enhancement in In-GaInSb superlattices Appl Phys Lett 1994 64 3160 3162 10.1063/1.111325

[B5] Rogalski A Material considerations for third generation infrared photon detectors Infrar Phys Technol 2007 50 240 252 10.1016/j.infrared.2006.10.015

[B6] Johnson JL Samoska LA Gossard AC Merz JL Jack MD Chapman GR Baumgratz BA Kosai K Johson SM Electrical and optical properties of infrared photodiodes using the InAs/GaInSb superlattice in heterojunctions with GaSb J Appl Phys 1996 80 1116 1127 10.1063/1.362849

[B7] Wei Y Bae J Gin A Hood A Jiang J Nah J Razeghi M Achyut K Dutta, Abdul Ahad S Awwal, Niloy K Type II InAs/GaSb superlattices for high-performance photodiodes and FPAs Proceedings of the Active and Passive Optical Components for WDM Communications III: September 8 2003; Orlando 2003 Dutta, Kazuo Fujiura: SPIE 501 511

[B8] Gunapala SD Ting DZ Ting CJ Hill CJ Nguyen J Soibel S Rafol SB Keo SA Mumolo JM Lee MC Liu JK Yang B Liao A Marija Strojnik Demonstration of 1Kx1K long-wave and mid-wave superlattice infrared focal plane arrays Proceedings of the Infrared Remote Sensing and Instrumentation XVIII: August 1 2010; San Diego 2010 Gonzalo Paez: SPIE 7808021 7808026

[B9] Hood A Evans AJ Ikhlassi A Sullium G Piquette E Lee DL Tennant WE Vurgaftman I Canedy CL Jackson EM Nolde JA Yi C Aifer EH Bjorn F Andresen, Gabor F Fulop, Paul R LWIR high performance focal plane arrays based on type-II strained layer superlattice (SLS) materials Proceedings of the Infrared Technology and Applications XXXVI: April 5 2010; Orlando 2010 Norton: SPIE 76601M1 76601M8

[B10] Rhiger DR Kvaas RE Harris SF Bornfreund RE Thai YN Hill CJ Li JV Gunapala S Mumolo JM Bjorn F Andresen, Gabor F Fulop, Paul R Progress with type-II superlattice IR detector arrays Proceedings of the Infrared Technology and Applications XXXVI: April 9 2007; Orlando Norton: SPIE 2007 654202

[B11] Yang QK Pfahler C Schmitz J Pletschen W Fuchs F Manijeh Razeghi, Gail J Trap centers and minority carrier lifetimes in InAs/(GaIn)Sb superlattice long wavelength photodetectors Proceedings of the Quantum Sensing: Evolution and Revolution from Past to Future: January 27 2003; San Jose 2003 Brown: SPIE 448 456

[B12] Rodriguez JB Plis E Lee SJ Kim H Bishop G Sharma YD Dawson JR Krishna S Bjorn F Andresen, Gabor F Fulop, Paul R Type-II InAs/GaSb strain layer superlattice detectors for high operating temperatures Proceedings of the Infrared Technology and Applications XXXVI: April 9 2007; Orlando 2007 Norton: SPIE 654208

[B13] Khoshakhlagh A Plis E Myers S Sharma YD Kirshna S Optimization of InAs/GaSb type II superlattice interfaces for long-wave infrared detection J Crystal Growth 2009 311 1901 1904 10.1016/j.jcrysgro.2008.11.027

